# Suicidal ideation, suicidal behaviors, and attitudes towards suicide of adolescents enrolled in the Alternative Learning System in Manila, Philippines—a mixed methods study

**DOI:** 10.1186/s41182-019-0149-6

**Published:** 2019-03-29

**Authors:** Crystal Amiel M. Estrada, Daisuke Nonaka, Ernesto R. Gregorio, Cynthia R. Leynes, Ronald T. del Castillo, Paul Michael R. Hernandez, Tatsuro Hayakawa, Jun Kobayashi

**Affiliations:** 1 Department of Global Health, Graduate School of Health Sciences, Faculty of Medicine, University of the Ryukyus, 207 Uehara, Nishihara-cho, Nakagami-gun, Okinawa, Japan; 2 Department of Environmental and Occupational Health, College of Public Health, University of the Philippines Manila, Manila, Philippines; 3 SEAMEO-TROPMED Regional Center for Public Health, Hospital Administration, Environmental and Occupational Health, Manila, Philippines; 4 Department of Health Promotion and Education, College of Public Health, University of the Philippines Manila, Manila, Philippines; 5 Department of Psychiatry and Behavioral Medicine, College of Medicine, University of the Philippines Manila, Manila, Philippines; 6 Department of Health Policy and Administration, College of Public Health, University of the Philippines Manila, Manila, Philippines; 7 Department of Psychiatry, Kohnodai Hospital, National Center for Global Health and Medicine, Ichikawa, Japan

**Keywords:** Suicidal ideation, Suicide, Attitude, Adolescent, (Alternative) education, Philippines

## Abstract

**Background:**

Globally, suicide is a significant cause of death among adolescents. Previous studies conducted in high-income countries suggest that students in alternative schools are more likely to engage in suicidal behaviors than those in formal schools. This study aimed to document suicidal ideation and behaviors among adolescent learners enrolled in the Alternative Learning System (ALS) in Manila, Philippines.

**Methods:**

A mixed methods study was conducted in 24 ALS centers in the city of Manila. ALS centers were stratified according to congressional district and selected using probability proportionate sampling. A cross-sectional survey to determine attitudes towards suicide and prevalence of suicidal ideation and behaviors was administered to 171 learners aged 13 to 17 years old. In-depth interviews with 18 teachers and 12 learners were conducted to explore the school psychosocial environment’s role on learners’ suicidal ideation and behaviors. Exploratory factor analysis was used to extract attitude factors. Fisher’s exact test and Student’s t-test were used to identify differences in sociodemographic characteristics and attitudes towards suicide between learners with or without suicidal ideation or behaviors. Qualitative data were analyzed using thematic analysis.

**Results:**

Non-specific active thoughts were the most common type of lifetime suicidal ideation (40.9%) while passive ideation was the most common in the past month (13.5%). Aborted suicide attempt was the most frequent behavior in both lifetime (16.4%) and in the past month (4.7%). Non-fatal suicide attempt in the past month was 2.3%, reaching 12.9% for the entire lifetime. Age, sex, education, and attitudes towards suicide were significantly associated with suicidal ideation or behavior. Thematic analysis showed five themes: (1) *fostering belongingness*, (2) *securing learners’ safety*, (3) *teaching philosophy*, (4) *teacher and learner beliefs towards suicidal behavior*, and (5) *availability of school-offered and community-based services*.

**Conclusion:**

Suicidal ideation and behaviors are prevalent among adolescent ALS learners. This study also showed a significant difference in attitudes towards suicide and sociodemographic characteristics between learners with and without suicidal ideation behaviors. It also suggests that the school psychosocial environment, through social norms and learner-teacher interactions, can potentially prevent progression of suicidal ideation to behavior, influence help-seeking, and promote mental health among learners.

## Background

Suicide is a global public health concern, accounting for approximately 1.5% of all deaths worldwide. Its prevalence is high among the adolescent and young adult populations [1]. In the Philippines, the prevalence of suicide is not clear since there is no country-wide suicide registry in place. However, the most recent Global School-based Student Health Survey (GSHS) reported that 11.6% of Filipino adolescents aged 13 to 17 years old considered attempting suicide while 16.8% attempted suicide at least once in the past year [2].

Suicidal ideation and behavior develop due to an interplay of factors. From Durkheim’s seminal work, many theories have been developed to better understand the development of suicidal ideation and behavior [3]. Bronfenbrenner’s ecological systems theory [4], which suggests that a social phenomenon develops through the interaction of the individual with its environment, has been applied to classify risk factors for suicide [5]. In recent years, research on how suicidal ideation progresses to suicidal behavior has also been gaining attention. The Integrated Motivational- Volitional (IMV) Model of Suicidal Behavior, one of the most recent models, describes the biopsychosocial context where suicidal ideation may develop, the factors that influence its occurrence, and the factors that influence the progression of ideation to behavior. Vulnerability (e.g., poverty) and stressful life events (e.g., early life adversity) constitute the milieu where suicidal ideation may develop. Feelings of defeat, humiliation, and entrapment also facilitate the development of suicidal ideation. Attitudes towards suicide can influence suicidal ideation. For example, more approving attitudes towards suicide were correlated with suicidal ideation among Slovene and Israeli adolescents [6, 7]. Finally, the transition from suicidal ideation to behavior occurs in the presence of moderators such as having access to means of suicide, increased capability to attempt suicide, exposure to suicide, and feelings of impulsivity [8].

Schools offer an excellent opportunity in implementing suicide intervention and prevention strategies since it is an institutional setting where children and adolescents spend most of their waking hours. Additionally, schools serve as a venue where adolescents interact with their peers, which in turn, can influence and create varied and unique psychosocial environments [9]. The school psychosocial environment which covers psychological (e.g., emotions and behavior) and social factors (e.g., teacher-student interactions) within the school [10] can influence adolescent mental health [11]. For example, a recent meta-analysis has shown that higher connectedness with school is associated with lower odds of suicidal ideation or behavior among school-going youth [12]. Such findings highlight the relationship of mental health outcomes and school features; however, the mechanisms through which the school environment influences adolescent suicidal ideation and behavior need to be further explored [13].

There is substantial published literature on suicidal ideation and behaviors among school-going adolescents and on developing effective school-based suicide prevention and intervention strategies. Yet, the majority are focused on formal education settings [14–18], and literature on alternative education programs is limited. Alternative education, or non-formal education, is defined by the United Nations Educational, Scientific and Cultural Organization International Standard Classification of Education (UNESCO ISCED) as an institutionalized education provided as an addition or an alternative to formal education. Since it aims to provide access to education for all, it is available to any individual regardless of age or ethnicity [19]. Students in alternative education are considered a vulnerable population since they live through risk factors both at the social and individual level which contribute to health disparities. In comparison with students attending regular high schools, students who are attending alternative high schools in the USA and New Zealand have been found to be at significantly higher risk for violence-related injury, multiple risk behaviors such as alcohol and tobacco use, and substance abuse, and suicide attempts [20–23]. A review of published literature on health behaviors and mental health of students attending alternative schools also found a similar pattern of high prevalence of high-risk behaviors; additionally, the review found less published literature reporting on social environmental variables and emotional or mental health outcomes [21].

In the Philippines, the Alternative Learning System (ALS) of the Department of Education is a parallel learning system which is an alternative to mainstream formal education. Its objective is to provide access to basic education to Filipino out-of-school youth and adults. While there are some published studies on the ALS in the Philippines, most are focused on education outcomes [24, 25]. According to program specialists from the Department of Education, information on the health status of learners enrolled in the ALS is limited. Furthermore, information on the mental health of ALS learners and how the school environment can affect learners’ suicidal ideation and behaviors have yet to be explored. To address the gaps in knowledge, this study was conducted to document suicidal ideation and behaviors among adolescent learners enrolled in the ALS. It specifically aimed to (1) determine the prevalence of suicidal ideation and behaviors among adolescent learners in the ALS, (2) describe the attitude of adolescent learners towards suicide, (3) assess the association between suicidal ideation and behaviors and participant characteristics (sociodemographic factors and attitudes towards suicide), and (4) explore the role of the ALS’ psychosocial environment on the suicidal ideation and behaviors of adolescents enrolled in the ALS.

## Methods

### Study design

A concurrent mixed methods study approach was utilized to develop a clearer and more comprehensive understanding of suicidal ideation and behaviors of adolescent learners enrolled in the ALS. This approach “*gathers both quantitative and qualitative data, integrates the two, and then draws interpretations based on the combined strengths of both sets of data to understand research problems*” [26]. A cross-sectional survey was employed to determine the prevalence of suicidal ideation and behaviors among adolescent learners and their attitudes towards suicide whereas grounded theory guided the qualitative methodology. Qualitative data were collected to extend the understanding of the learners’ suicidal ideation and behaviors and their attitude towards suicide by exploring the role of the school psychosocial environment on the suicidal ideation and behaviors of the adolescent learners.

### Study setting and participants

The study was conducted in ALS centers in the city of Manila, the capital city of the Philippines. There were 98 ALS centers subdivided into six congressional districts: 19 schools in District 1, 19 schools in District 2, 14 schools in District 3, 18 schools in District 4, 12 schools in District 5, and 17 schools in District 6. Of these centers, eight did not have an ALS teacher or instructional manager, while four did not have records in the online learner registration database and were therefore excluded from the sampling frame. More than half of the 6000 learners were males (65.6%), and 35.8% were aged 13 to 17 years old. Majority of the learners were enrolled in the Secondary Level Accreditation and Equivalency (A&E) program—an educational qualification comparable to the junior high school qualification of the formal school system. The city was selected since no previous study focusing on the ALS environment has been conducted. Permission to conduct the study was granted by the Department of Education Manila Division Office and the respective school principals. Ethical approval of the study was granted by the Ethical Committee for Medical and Health Research Involving Humans, University of the Ryukyus, Japan (Protocol Identification Number 1247) and the National Children’s Hospital – Institutional Review Board, Philippines (NCH – IRB 2017-24-NCT-01).

The sample size for the survey was computed using the reported prevalence of suicidal behavior (16.8%) in the 2015 Global School-based Health Survey [3]. With a 95% confidence level, 5% total width of confidence interval, a design effect of 2.0, and a possible 10% non-response rate, the sample size was determined to be 476. Participants were selected using a stratified cluster sampling wherein the ALS centers in the city of Manila were stratified according to school district and four ALS centers from each district were selected using probability proportionate sampling. Adolescent learners aged 13 to 17 years old who were enrolled in the ALS Accreditation and Equivalency (A&E) program of the selected 24 ALS centers in the city of Manila were invited to participate. Adolescent learners who have intellectual disability as reported by the ALS teacher were excluded from the study. To minimize non-response, researchers visited each ALS center at least four times to conduct the survey. Among the learners who participated in the survey, only those who had been studying for at least 3 months in the ALS at the time of the study were interviewed. Additionally, teachers assigned to the ALS centers included in the survey were also interviewed.

### Quantitative data collection

The prevalence of suicidal ideation and behaviors was measured using the English version of the Columbia Suicide Severity Rating Scale (C-SSRS). It can identify whether an individual has thought about suicide, if they have engaged in preparatory acts or behavior for suicide, or whether they have engaged in suicidal behavior. Suicidal behaviors were categorized as follows: an attempt to commit suicide but was stopped by someone or something was classified as an *interrupted attempt*; an attempt stopped by the individual themselves before actually engaging in any self-destructive behavior was an *aborted attempt*; any behavior related to taking steps towards making an attempt was considered a *preparatory act or behavior*; and a potentially self-injurious act committed with at least some intent to die was considered a *nonfatal actual attempt*. The scale has demonstrated convergent, divergent, and predictive validity in three multi-site studies conducted among adolescent suicide attempters, adolescents with depression, and adults with a history of recent suicide attempts and non-suicidal self-injurious behavior. Additionally, when compared with the Columbia Suicide History Form and ratings from an independent suicide evaluation board, it demonstrated high specificity and sensitivity in identifying aborted, interrupted, and actual suicide attempts among American adolescent attempters [27]. In the present study, the scale was administered through face-to-face interviews by researchers who completed online training provided by the developers of the scale and a supplemental training conducted by a child and adolescent psychiatrist [CL] in the Philippines.

Attitudes towards suicide were measured using the Attitudes Towards Suicide Scale (ATTS) [28]. The scale is composed of 37 items answerable by a 5-point Likert scale with responses ranging from “strongly agree” to “strongly disagree.” It has been found to be feasible and valid [29], and its psychometric properties have been validated and employed in a wide range of populations [30–32]. In the present study, the ATTS was translated into Filipino using a forward and backward translation process. The forward translation was developed by the researcher and pre-tested among learners aged 13 to 17 years old. Aspects of the translation which were unclear and ambiguous were then revised. Finally, the draft was translated back into English by an independent psychologist and a linguist. The translators and researchers then compared the back-translated version with the original version. Unclear or differences in the back translations were then discussed and incorporated into the draft [33]. The face validity of the final translated version was then tested among ALS learners in Quezon City, a nearby city.

### Quantitative data analysis

Survey responses were double encoded, and quantitative data analysis was carried out using Statistical Package for the Social Sciences (SPSS) version 21. Descriptive statistics were calculated for sociodemographic characteristics of respondents. Fisher’s exact test was used to determine if there was a significant difference in sociodemographic characteristics between learners who have or do not have suicidal ideation or behaviors. Kaiser-Meyer-Olkin (KMO) Measure of Sampling Adequacy and Bartlett’s Test of Sphericity were conducted to assess the suitability of data for factor analysis. Previous studies on attitudes towards suicide yielded different factorial structures; hence, exploratory factor analysis was conducted to identify factors applicable to the Filipino setting [28, 30–32]. Exploratory factor analysis using principal axis factoring and varimax rotation was conducted to extract factors pertaining to the learners’ attitudes towards suicide. Seven out of the 37 items of the ATTS scale were not included in the factorization due to floor and ceiling effects observed in the responses. Factors with Cronbach’s alpha scores lower than 0.70 were not included in the analysis [34]. Factor scores were generated using the regression method. Student’s t-test was used to determine significant differences in factor scores and disclosure of suicidal ideation or behavior. All statistical tests were conducted with a significance level set at *p* <  0.05.

### Qualitative data collection

In-depth interviews focusing on teachers’ and learners’ experiences in the ALS, their interaction with each other, and sources of information on mental health promotion and suicide prevention within the school were conducted. The interview guide was pretested among ALS learners in Quezon City. The interviews were held either at the guidance counselor’s office or in a separate area of the classroom away from other learners to ensure privacy. All interviews were conducted in a mix of English and Filipino languages. The interviews were recorded using a digital voice recorder with the permission of the respondents, and all audio recordings were transcribed by a research assistant. Transcription guidelines were developed by the researchers and used in transcribing the interviews to ensure respondent anonymity and accuracy and consistency of all transcripts.

### Qualitative data analysis

Digital audio recordings of the interviews were transcribed into Microsoft Word documents. Data analysis was undertaken by reading through the transcriptions, coding the data, and developing themes. Saldana’s coding methods were followed to fragment data [35]. Codes were generated through the process of having two researchers code several transcripts independently, compare the codes generated, create a definition for each, and use the codes for subsequent transcripts. Coding and analysis were also conducted in consultation with other co-authors. Thematic analysis, which identified patterns within the data, was used to classify the categories into themes [36].

## Results

### Sociodemographic characteristics of the ALS learners

**Fig. 1:**
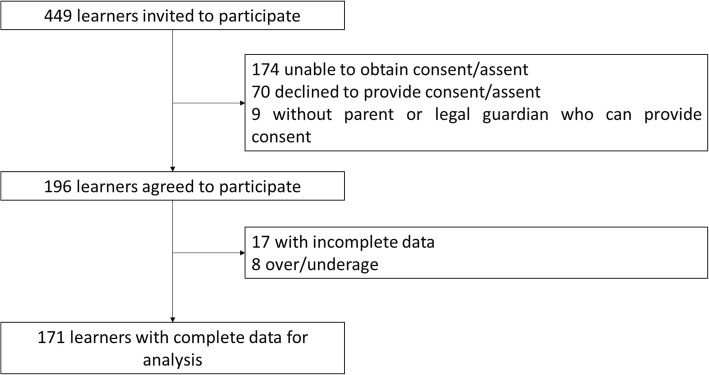
Flow of participant inclusion in the study

Of the 449 learners invited to participate, 70 declined to provide consent or assent, nine had no parent or legal guardian who can provide consent, and 174 learners were unable to obtain consent from parents or legal guardians. A total of 196 learners agreed to participate which yielded a 43.7% participation rate. After removing 17 learners which had incomplete data and eight which were over or under the age limit, data from 171 learners were included for data analysis (Fig. 1).

Majority of the respondents were male (66.1%), single (89.5%), unemployed (85.4%), and Roman Catholic Christians (82.5%) (Table 1). Majority of the respondents were aged 16 (39.2%) to 17 years old (33.3%). More than half of the respondents did not finish secondary school (57.3%), with family problems being the most common reason for leaving school (49.1%).

**Table 1:** Sociodemographic characteristics of surveyed ALS learners from 24 selected ALS centers in the city of Manila, Philippines (*n* = 171)

Variable	Frequency	Percentage
Sex		
Male	113	66.1
Female	58	33.9
Age		
13	3	1.8
14	15	8.8
15	29	17.0
16	67	39.2
17	57	33.3
Civil status		
Single	153	89.5
Cohabiting	8	4.7
Others	10	5.8
Religion		
Roman Catholic	141	82.5
Islam	8	4.7
Others	22	12.9
Highest educational attainment		
Primary school undergraduate	40	23.4
Primary school graduate	29	17.0
Secondary school undergraduate	98	57.3
Others	4	2.4
Employment status		
Employed	25	14.6
Unemployed	146	85.4
Reason for leaving school^a^		
Family problem	84	49.1
Family cannot afford school	31	18.1
Moved away	23	13.5
No interest	21	12.3
Sickness/disability	19	11.1
To work/to find work	15	8.8
School is far	11	6.4
Others	25	14.6

^a^Multiple answers allowed

### Prevalence of suicidal ideation and suicidal behavior

Non-specific active suicidal thoughts were the most common type of lifetime suicidal ideation (40.9%) (Table 2). Passive ideation, characterized by the question about wishing to be dead, was the most frequently reported type of suicidal ideation in the past month (13.5%). Aborted suicide attempt had the highest proportion among all types of behavior measured, with 16.4% and 4.7% of respondents reporting an aborted attempt in their lifetime and in the past month, respectively.

**Table 2:** Proportion of surveyed ALS learners with lifetime and past month suicidal ideation and suicidal behavior (*n* = 171)

Event	Lifetime		Past month	
	*n*	% (95% CI)	*n*	% (95% CI)
Suicidal ideation				
Wish to be dead	69	40.4 (33.0–47.8)	23	13.5 (8.4–18.6)
Non-specific active suicidal thoughts	70	40.9 (33.5–48.3)	16	9.4 (5.0–13.8)
Active suicidal ideation with any methods (not plan) without intent to act	40	23.4 (17.1–29.7)	12	7.0 (3.2–10.8)
Active suicidal ideation with some intent to act, without specific plan	27	15.8 (10.3–21.3)	6	3.5 (0.7–6.3)
Active suicidal ideation with specific plan and intent	26	15.2 (9.8–20.6)	5	2.9 (0.4–5.4)
Suicidal behavior				
Aborted attempt	28	16.4 (10.9–21.9)	8	4.7 (1.5–7.9)
Non-fatal suicide attempt	22	12.9 (7.9–17.9)	4	2.3 (0.1–4.5)
Preparatory acts or behavior	13	7.6 (3.6–11.6)	4	2.3 (0.1–4.5)
Interrupted attempt	11	6.4 (2.7–10.1)	4	2.3 (0.1–4.5)

Females were more likely to have passive suicidal ideation and non-specific active suicidal thoughts than males (Table 3). Learners aged 16 to 17 years old were more likely to have active suicidal ideation with specific plan and intent when compared to the younger cohort. Conversely, no significant differences in suicidal ideation were observed between learners who reached primary school only and those who reached secondary school. However, learners who reached secondary school were more likely to have a non-fatal suicide attempt than those who reached primary school only.

**Table 3:** Comparison of surveyed learners with suicidal ideation and behaviors according to sex, age, and highest educational attainment

Event	Sex			Age			Highest educational attainment		
	Male *n* (%)	Female *n* (%)	*p* value	13 to 15 years old *n* (%)	16 to 17 years old *n* (%)	*p* value	Up to primary level only *n* (%)	Up to secondary level *n* (%)	*p* value
Suicidal ideation									
Wish to be dead	34 (30.1)	35 (60.3)	< .001	16 (34.0)	53 (42.7)	0.383	25 (36.2)	44 (43.1)	0.428
Non-specific active suicidal thoughts	40 (35.4)	30 (51.7)	0.049	14 (29.8)	56 (45.2)	0.082	22 (31.9)	48 (47.1)	0.058
Active suicidal ideation with any methods (not plan) without intent to act	24 (21.2)	16 (27.6)	0.446	8 (17.0)	32 (25.8)	0.312	11 (15.9)	29 (28.4)	0.067
Active suicidal ideation with some intent to act, without specific plan	15 (13.3)	12 (20.7)	0.268	3 (6.4)	24 (19.4)	0.058	7 (10.1)	20 (19.6)	0.134
Active suicidal ideation with specific plan and intent	16 (14.2)	10 (17.2)	0.655	2 (4.3)	24 (19.4)	0.016	6 (8.7)	20 (19.6)	0.054
Suicidal behavior									
Aborted attempt	15 (13.3)	13 (22.4)	0.133	7 (14.9)	21 (16.9)	0.821	10 (14.5)	18 (17.6)	0.676
Non-fatal suicide attempt	10 (8.8)	12 (20.7)	0.051	1 (2.1)	21 (16.9)	0.293	4 (5.8)	18 (17.6)	0.034
Preparatory acts or behavior	6 (5.3)	7 (12.1)	0.134	2 (4.3)	11 (8.9)	0.519	3 (4.3)	10 (9.8)	0.246
Interrupted attempt	8 (7.1)	3 (5.2)	0.752	1 (2.1)	10 (8.1)	0.293	5 (7.2)	6 (5.9)	0.758

Multiple suicide attempts were common and observed in both males and females (Table 4). Aborted attempt was the most frequent type of attempt, with a total of 19 single attempts and 13 attempts committed more than two times. Furthermore, the total number of multiple attempts was also higher than single attempts.

**Table 4:** Number of lifetime and past month aborted attempt, interrupted attempt, and non-fatal suicide attempt among surveyed ALS learners according to sex

Type of attempt	Lifetime		Past month	
	Male	Female	Male	Female
Aborted				
Once	8	6	2	3
Twice	2	1	0	0
More than two times	5	6	1	1
Interrupted				
Once	4	1	1	2
Twice	1	1	0	0
More than two times	3	1	0	1
Non-fatal				
Once	4	4	1	1
Twice	3	2	0	0
More than two times	3	6	0	2
Total	33	28	5	10

### Attitudes towards suicide

Initial factor analysis using principal axis factoring of the 30 items indicated an 11-factor solution explaining 62.8% of the total variance. However, the scree plot, eigenvalues, parallel analysis, and factor loadings showed a three-factor solution which explained 22.0% of the variance (Table 5). Factor 1 showed an eight-item structure composed of items 5, 16, 20, 21, 24, 34, and 36 which explained 8.8% of the variance. Factor loadings ranged from 0.41 to 0.59. Cronbach’s alpha was 0.73. Factor 1 reflected permissive or accepting attitudes towards suicide, as demonstrated by item 5: “Suicide is an acceptable means to terminate an incurable disease.”

**Table 5:** Obtained factors, explained variance, items, factor loadings, and internal consistency of the ATTS

Factor	Factor loading	Explained variance	Internal consistency
1. Suicide is acceptable		8.8%	0.73
People do have the right to take their own lives	0.59		
Suicide is an acceptable means to terminate an incurable disease	0.58		
I can understand that people suffering from a severe, incurable disease commit suicide	0.54		
I would consider the possibility of taking my life if I were to suffer from a severe, incurable disease	0.51		
If someone wants to commit suicide it is their business and we should not interfere	0.46		
There may be situations where the only reasonable solution is suicide	0.44		
A person, once they have suicidal thoughts, will never let them go	0.42		
I would like to get help to take my own life if I were to suffer from a severe, incurable, disease	0.41		
2. Suicide is a process		8.6%	0.70
Loneliness could for me be a reason to take my life	0.60		
It is mainly loneliness that drives people to suicide	0.59		
Most suicide attempts are caused by conflicts with a close person	0.55		
There is a risk of evoking suicidal thoughts in a person’s mind if you ask about it	0.49		
When a person commits suicide it is something that he/she has considered for a long time	0.43		
Usually relatives have no idea about what is going on when a person is thinking of suicide	0.41		
Suicide happens without warning	0.41		
3 Suicide is incomprehensible		4.6%	0.42
On the whole, I do not understand how people can take their lives	0.54		
People who make suicidal threats seldom complete suicide	0.42		

Factor 2 showed a seven-item structure composed of items 10, 11, 14, 22, 25, 28, and 35 which explained 8.6% of the variance. The factor loadings ranged from 0.41 to 0.60, and the Cronbach’s alpha was 0.70. Factor 2 included statements which acknowledged suicide as a process, as demonstrated by item 10: “When a person commits suicide it is something that he/she has considered for a long time.”

Factor 3 was not included in the analysis because of low Cronbach’s alpha score (0.42).

Learners who answered “yes” to the question on (1) wish to be dead, (2) active suicidal ideation but without intent to act, or (3) active suicidal ideation with some intent to act but no specific plan held more permissive attitudes than those who answered “no” (Table 6). Learners who engaged in aborted or interrupted attempts also held more permissive attitudes than those who did not. No significant difference in terms of permissiveness towards suicide was observed between those who reported suicidal ideation or behavior and those who did not. In contrast, except for those who reported an aborted attempt, learners who experienced any type of suicidal ideation and engaged in suicidal behavior in their lifetime were significantly more agreeing that suicide is a process as compared to those who did not.

**Table 6:** Independent Sample t-test for mean factor scores on learners’ attitudes towards suicide and lifetime suicidal ideation and behaviors

	Presence of ideation/behavior		Absence of ideation/behavior		*p* value
	Mean	SD	Mean	SD	
Factor 1: Permissiveness towards suicide					
Suicidal ideation					
Wish to be dead	0.001	0.857	− 0.001	0.863	0.990
Non-specific active suicidal thoughts	− 0.086	0.877	0.059	0.844	0.278
Active suicidal ideation with any methods (not plan) without intent to act	0.006	0.860	− 0.002	0.861	0.957
Active suicidal ideation with some intent to act, without specific plan	0.054	0.930	− 0.010	0.847	0.724
Active suicidal ideation with specific plan and intent	− 0.127	0.917	0.023	0.849	0.415
Suicidal behavior					
Preparatory acts or behavior	− 0.370	0.816	0.030	0.860	0.106
Aborted attempt	0.011	0.875	− 0.002	0.858	0.940
Interrupted attempt	0.074	0.855	− 0.005	0.861	0.769
Non-fatal suicide attempt	− 0.183	1.004	0.027	0.835	0.286
Factor 2: Suicide as a process					
Suicidal ideation					
Wish to be dead	− 0.314	0.779	0.212	0.834	< 0.001
Non-specific active suicidal thoughts	− 0.320	0.780	0.222	0.829	< 0.001
Active suicidal ideation with any methods (not plan) without intent to act	− 0.374	0.766	0.114	0.844	0.001
Active suicidal ideation with some intent to act, without specific plan	− 0.441	0.817	0.083	0.833	0.003
Active suicidal ideation with specific plan and intent	− 0.605	0.770	0.109	0.820	< 0.001
Suicidal behavior					
Preparatory acts or behavior	− 0.850	0.632	0.070	0.829	< 0.001
Aborted attempt	− 0.244	0.803	0.048	0.854	0.097
Interrupted attempt	− 0.627	0.853	0.043	0.835	0.011
Non-fatal suicide attempt	− 0.561	0.808	0.083	0.830	0.001

### Qualitative results

A total of 12 learners and 18 teachers were interviewed about their experiences in the ALS, their interaction with each other, sources of mental health promotion and suicide prevention information and services within the school. Teachers were also further probed about their experience of having a learner who disclosed suicidal ideation or behavior and how they managed the situation. On the other hand, learners were also interviewed about their basic knowledge on suicide prevention. Of the 18 teachers, 13 were females and five were males. The teachers’ years of teaching experience in the ALS ranged from less than a year to 20 years. Eleven teachers reported that they had learners who disclosed suicidal ideation and/or behavior. Seven of the 12 learners interviewed were males, and five were females. Of the 12 learners, nine disclosed suicidal ideation and/or behavior.

Of the interviews conducted, 83 codes were generated, which were then sorted into 15 categories. Ultimately, five themes emerged from the data: (1) *fostering belongingness*, (2) *securing learners’ safety*, (3) *teaching philosophy*, (4) *teacher and learner beliefs towards suicidal behavior*, and (5) *availability of school-offered and community-based services*. The meanings arising from the qualitative data highlighted the role of the school environment on learners’ suicidal ideation and behavior.

#### Fostering belongingness

One of the key concepts which emerged from the study is the sense of belongingness. The learners reported that the sense of having a family within or belonging to the “ALS family” was emphasized in the classroom. For example, one learner said:

*“They treat me like their own sibling, because – we are like a whole family here.”* (Male Learner, 17 y/o)

This was exemplified by teachers taking on the role of being a second parent or an elder sibling to the learners and the following teacher illustrates this relationship:

*“So what I did was I gave them whatever they needed. What they didn’t have. What they were searching for in their own families, I gave to them. What I did was if they needed a mother, I am here. I also told them, ‘As your mother, I will take care of you no matter what happens. And I will make sure that you will graduate’.”* (Female Teacher)

The learners also talked about the availability and responsiveness of their teachers to their needs for comfort or support in times of problems, either face to face or through social media. Teachers corroborated that there are group chats or messages in social media or through short message service (SMS) for their class to make sure that they can reach out to learners and vice versa. Aside from these, several teachers also reported that they welcomed the learners to their own homes, extending their teaching sessions during weekends and allowing students to visit their own private homes.

Another key meaning expressed in this theme is the support and motivation the learners received from both their teacher and fellow learners.

*“She’s [the teacher] there, ready to help with anything. She’ll explain it clearly even if you already feel mad – she extends her patience.” *(Male Learner, 17 y/o)

Teachers consistently gave encouragement to the learners by praising their works and efforts and motivating them to do better. When learners had personal problems, teachers also provided encouragement and advice to the learners. The following quote from a teacher highlights this:

*“I’m here if they need someone to talk to. If they need a shoulder to cry on, I’m also here. I always ask them how they’re doing. I always give them a tap on the back, saying “That’s great.” I encourage them so that they can have hope for themselves. I am teaching them to dream.”* (Female Teacher)

Learners were also able to provide support and motivation to their fellow learners. Learners discussed their personal problems within their circle of friends. For example, learners who already passed the Accreditation and Equivalency exam comforted and encouraged the learners who failed. One learner highlighted these words of support he received from fellow learners:

*“They told me, ‘Don’t worry, we’re here, we have your back. No matter what happens you have our support’.”* (Male Learner, 17 y/o)

While there are positive benefits to having support and motivation among the groups formed by the learners, it also poses some negative effects. For example, teachers reported that learners engage in risky health behaviors such as alcohol use. Oftentimes, learners discuss their personal problems while having a drinking session and this practice is identified by the teachers as an opportunity to encourage risky health behaviors within groups. Another negative effect is verbal oppression among learners. Teachers talked about some learners labeling fellow learners with offensive nicknames pertaining to their physical appearance or sexual orientation, which lead to disagreements within the classroom. One learner mentioned his experience of being ridiculed by others: *“They will tease you. They will tell you, ‘You’re gay!’”*. (Male learner, 17 y/o)

#### Securing learners’ safety

Another theme which emerged from the data is how both the teacher and fellow learners secured learners’ safety. Key strategies to prevent learners from engaging in or developing suicidal behavior included preventing risky health behaviors such as alcohol abuse and identifying and acting on warning signs among learners, such as social media posts hinting about suicide. Some teachers reported seeing learners posting messages with hints about suicide in social media sites; thus, they communicated with the learners immediately to ensure their safety:

*“…even posted in Facebook. He cut his wrist. I called him immediately. I didn’t even reply or comment on Facebook. I called him.” *(Female Teacher)

Response measures, on the other hand, ranged from removing learners from dangerous situations or environment, diverting learners’ attention from suicidal thoughts, and monitoring learners with suicidal behaviors through constant follow-ups or calls. One teacher described her action as letting one learner stay in her house to watch over and remove her from the stressful environment that triggered suicidal thoughts. Strategies to monitor learners who disclosed suicidal behavior included teachers asking other learners to watch over the learner and to report any possible emergency, calling learners on the phone, or sending messages through social media to make sure they are safe. Learners, on the other hand, diverted the attention of their fellow learners from their suicidal thoughts by accompanying them to public places like malls. One learner, as a response to a classmate who disclosed suicidal behavior, removed any item or material which his classmate can use to hurt himself:

*“So what I did was to take away from him things which he can use to hurt himself. Also, when we were still in Catungba [codename for learners’ city], I used to keep him company frequently to cheer him up. Sometimes I go out with him even if I don’t have money; I would find a way just so he wouldn’t think about suicide.” *(Male Learner, 17 y/o)

#### Teaching philosophy

Teachers’ values and beliefs related to teaching and how it related to learners with suicidal behavior form the theme *teaching philosophy*. Teachers’ beliefs and how they interacted with learners were the key meanings that emerged in this theme.

Many teachers talked about extending their role as a teacher beyond teaching; that is, they also need to provide care and love for the learners. Many teachers talked about acting as a counselor to the learners, as exemplified by a teacher’s statement:

*“We ALS teachers serve as teacher, counselor, coordinator, then we also do the surveys”.* (Female Teacher)

How the teachers interacted with the learners was also influenced by their teaching philosophy. Most of the teachers reported that they used a friendly but authoritative approach towards learners; that is, the teachers befriended learners, developed a close relationship with them, but still maintained the student-teacher boundary. To develop these meaningful relationships with the learners, teachers realized that they needed to relate to the learners and “enter their world”. Learners recognize their teachers’ efforts to connect and show their appreciation as exemplified by this student’s experience:

*“Ma’am was here yesterday and we were eating together, she really listens to each of our stories. Sometimes even if there’s no classes on Saturday she’s still here. Sometimes when we’re at home, we think “It’s really fun in school because Ma’am is there”. She really relates to us. Yes, we are really close to Ma’am.”* (Male Learner, 17 y/o)

Furthermore, most of the teachers stressed the importance of serving as a role model to the learners. They emphasized their responsibility to teach by example; for example, in order for them to teach about respect, they treated learners with respect. Another value that the teachers talked about was promoting learner independence. By allowing learners to talk about their problems freely and openly, the teachers gave the learners freedom to make their own decisions. Through this process, the teachers guided the learners so that they can decide for themselves and analyze the positive and negative implications of their decisions:

*“It’s more like we shouldn’t dictate what the learner should do; it’s more on to sympathize and give encouraging words. We won’t say, ‘oh, this is your problem, this is what you should do’. What we were instructed was to let the learner decide what he or she should do for his or herself.”* (Female Teacher)

Connected with this, they also taught the learners the value of being self-reliant; that is, the teachers developed learners’ ability to survive on their own. They emphasized the need for the learners to learn how to help themselves and not just rely on parents or teachers “spoon-feeding” them:

*“I tell them, ‘You cannot always rely on other people. If you want a good future, you have to be able to help yourself. Because, uh, it’s not like someone will be there to help you for all of your life’.”* (Female Teacher)

Religious and spiritual beliefs were also key meanings in this theme, especially in addressing learners’ suicidal behavior. Turning to religious practices such as praying was reported by teachers when addressing learners who disclosed suicidal thoughts or behaviors. Most teachers also talked about giving religious or spiritual advice to learners when they sought advice or disclosed personal problems or suicidal thoughts:

*“So – really the advice I tell them is that their life is a gift. We do not have the right to take it or end it by our own hands. Only God has the right to take it from us.” *(Female Teacher)

Teachers also integrated spiritual or religious development in class or as part of extracurricular activities. By incorporating religious and spiritual development, teachers believed that learners gained comfort from their problems or suicidal behavior. However, teachers also emphasized respect for learners’ individual religious differences:

*“I also consider their individual differences when it comes to religion. But I also emphasize that even if we have different religions, we all still have a God. Our God does not sleep. Our God loves us.”* (Female Teacher)

#### Teacher and learner beliefs towards suicidal behavior

How teachers and learners alike perceived suicidal behavior also played a role in the school psychosocial environment. Both teachers and learners alike believed that suicidal behavior is influenced by several factors such as biological and psychosocial characteristics. For example, one learner stated: *“Caused by – uh – extreme stress, problem, or mental disorder.”* (Male Learner, 17 y/o)

Teachers believed that suicidal behavior is rooted from biological or physiological factors, such as an imbalance in mental faculties or underlying mental disorders. Furthermore, teachers believed that suicidal behaviors must have physical manifestations. One teacher believed that a learner with suicidal ideation or behavior must “manifest sadness at one point” while another teacher could not believe that one learner disclosed suicidal behavior since the learner was an exemplary student in class. Aside from stress and family problems, teachers also emphasized the role of social media on the development of suicidal ideation and behaviors among learners. One teacher stated that being ridiculed or embarrassed in social media can drive a learner to have suicidal thoughts or engage in suicidal behaviors. Some teachers also believed that suicide is a call for help; however, some also endorsed the belief that suicide is a “family issue”; that is, teachers cannot intrude or intervene since it is something that the family has authority over.

Religious beliefs also influenced how teachers and learners perceived suicidal behaviors. Teachers and learners alike subscribed to the belief that suicidal behavior is against or contrary to the teachings of their religion—committing suicide is a sin against God. Fear of going to hell because of committing suicide was also consistently mentioned by teachers and learners alike, with learners specifying this as a reason why they have not engaged in suicidal behavior. One learner elaborated:

*“When you commit suicide, it’s a sin against God. For example, if you commit suicide, you won’t go to heaven. You’ll go straight to hell.”* (Male Learner, 17 y/o)

Furthermore, some teachers believed that their faith is a strength in addressing learners’ suicidal behaviors. One teacher reported that their own spiritual strength helps or can help in managing learners with suicidal behavior, stating, *“It’s not difficult for me. It’s not difficult because I have – I serve in the Church.” *(Male Teacher)

Negative beliefs and attitudes towards suicide were also reported by the respondents. Some learners believed that fellow learners who engage in suicidal behaviors are not in their right mind, with one even describing them as: *“Oh, this one must be crazy. Committing suicide.”* (Male Learner, 17 y/o)

Some teachers believed that the reasons for learners engaging in suicidal behavior, such as failed romantic relationships or failing in exams, were “minor problems” and does not justify the behavior. Some reported that it is cowardice on part of the learners for not facing their problems and engaging in suicidal behavior instead. Some teachers felt uncertain whether to take learners’ disclosure of suicidal behavior seriously and could not ascertain whether learners were joking when they talked about suicide. Nevertheless, the teachers stressed the importance of helping learners regardless of their own perception of the gravity of reasons they have for engaging in suicidal behaviors.

#### Availability of school-offered and community-based services

The availability of school-offered and community-based services also emerged as a theme in this study. School- offered services such as the availability of school staff, health-related modules, and sponsored events and community-based services such as religious-oriented and government programs are key concepts in this theme.

The teachers reported that learners mostly rely on them for mental health information but other school staff such as the school nurse, school guidance counselor, or teacher are also possible sources of information. However, learners identified the ALS teacher as the only school personnel who they will consult if they need information or help on such topics.

When managing learners with suicidal ideation or behaviors, ALS teachers reported that they are the front liners—that is, they try to handle the case as much as they can. Whenever the ALS teacher needs the assistance of other school staff to address learners’ suicidal ideation and behavior, they identified the guidance counselors or guidance teachers or the school nurses and doctors as the next point of referral. However, there were variations in the referral structure. Some teachers reported that after the guidance counselor is notified, the case will be referred to the school principal, while others reported that the school nurse will be notified. Others also reported that the case will be reported to external offices or a government agency such as the Department of Social Welfare and Development if management within the school cannot resolve the case. Teacher concerns that the school guidance counselor or teacher may face challenges addressing the ALS learners’ needs due to their already heavy workload in the formal school or their limited technical qualification also emerged during the interviews.

The ALS teachers identified the need to strengthen their skills on interacting with learners with possible suicidal ideation or behavior, especially since they are the first person that learners consult whenever they have concerns. One specific example of skill that teachers identified is the need to develop mentoring or life coaching skills to increase learners’ morale and self-esteem. Additionally, interacting with and giving advice to learners with suicidal behaviors was another identified need, since they had limited specific or formal training on mental health and management of learners with suicidal behaviors:

*“I want to learn how to interact with them, especially when they tell you they are thinking about suicide. As much as possible, I will give advice up to the extent that I can. But it’s better if I have adequate training. I can give them better advice.”* (Female Teacher)

Aside from developing their skills, teachers also talked about the importance of having support from mental health experts who can conduct seminars or trainings on how to address or prevent suicide among learners.

The availability of health-related modules is another key meaning in this theme. Some learning modules being used in the ALS indirectly discuss suicide prevention. Teachers identified modules that address risk factors such as substance abuse while others discuss protective factors such as resolving conflicts, handling stress and exploring one’s self. Talking about a module, one participant explained:

*“There’s a part of that a person gets depressed – there’s a part there that says they want to commit suicide, but it should be that if you have a problem in life you should not take it too seriously. Things like that”* (Male Learner, 17 y/o)

By discussing the module about protective factors in class, one teacher noticed changes in learners, stating that:

*“Their emotions…and their lifestyle…and how they interact with others changed. They also learned how to handle their problems, unlike before that they would consult me even for the smallest problem.” *(Female Teacher)

One interesting finding in this study is the identification of livelihood or practical skills as a contributor to improved well-being. Teachers perceived that the livelihood skills help improve the learners’ financial situation, which leads to an improved emotional state. Moreover, the livelihood skills learned in the ALS provide learners with a sense of purpose that diverts their thoughts from suicide:

*“Of course, their mind will be busy. They will be active. Instead of just loitering around, thinking of depression, their family problems – those trigger them to commit suicide”* (Female Teacher)

The teachers also talked about health seminars sponsored by the Department of Education for the learners and their parents annually. Previously covered topics include HIV awareness and prevention and substance abuse. However, most teachers reported that there has been none focused on mental health or suicide prevention.

Aside from school-offered services, community-based services were also available to the learners. Learners were involved in church ministries and activities invited by the ALS teachers, such as youth cell groups. Pastors from church organizations also provide counseling for learners. This was exemplified by one of the learners’ experience:

*“My Church leader, I told him a lot of my problems at home – in life. He told me a lot of things about that – problems. He told me not to do it because it’s bad.”* (Male Learner, 17 y/o)

Attending the church activities had positive effects on learners’ behavior and outlook in life. As one learner shared, his participation in church activities has helped him become a “better person”:

*“After I attended Church, I really became better. I learned how to communicate properly, and, I detached myself from a lot of [negative] things.”* (Male Learner, 17 y/o)

Aside from school services and religious-oriented services, other government agencies also provided services to learners and their families. Many learners are recipients of the conditional cash transfer program (locally known as 4Ps) of the Philippine government and the families receive social assistance and family development sessions as part of their enrolment in the program. Under the family development sessions, families receive classes on topics such as responsible parenthood and mental health. One ALS teacher described the coordination of services provided to the learners and their families:

*“The 4Ps require the families to visit the doctor, in the clinic. They have to visit the clinic regularly. Not just for illnesses, but the DSWD (Department of Social Welfare and Development) has to see their record. They have to visit the clinic to show proof to the DSWD that they have a record…Schools provide attendance and educational [sic], the clinic provides the health. The DSWD provides cash and services.”* (Male Teacher)

## Discussion

Using concurrent mixed methods, the present study documented suicidal ideation and behavior in the ALS. Non-specific active thoughts was the most common type of lifetime suicidal ideation (40.9%) while passive ideation was the most common in the past month (13.5%). Aborted suicide attempt was the most frequent behavior in both lifetime (16.4%) and in the past month (4.7%). Age, sex, education, and attitudes towards suicide were significantly associated with suicidal ideation or behavior. On the other hand, thematic analysis identified five themes how the school environment could influence learners’ suicidal ideation and behaviors: **(**1) fostering belongingness, (2) securing learners’ safety, (3) teaching philosophy, (4) teacher and learner beliefs towards suicidal behavior, and (5) availability of school-offered and community-based services.

Consistent with previous literature, the prevalence of suicidal ideation and behaviors among ALS learners was higher compared to learners in formal schools [20]. The prevalence of lifetime suicidal ideation in the present study was higher compared to learners studying in formal schools in the Philippines (11.6%). However, the prevalence in the formal school was based on the past 12 months rather than lifetime [2]. Moreover, the 16.4% lifetime prevalence of suicidal behavior (aborted attempt) among the surveyed ALS learners in this study is higher compared to learners enrolled in the formal high schools in the Philippines (7.9%) and Thailand (4.2%) [37]. The prevalence of suicidal behavior in this study was similar compared to a study on adolescents enrolled in US alternative high schools, wherein the reported prevalence of suicide attempt was 17.6% [21]. In terms of sex, females had significantly higher prevalence of suicidal ideation than males in this study. This finding is similar to a previous study conducted in junior high schools in Indonesia and Philippines wherein females are more likely to consider committing suicide [38]. In relation to age, the findings of the study corroborate a previous study conducted among Norwegian adolescents, wherein suicidal ideation was less frequent among adolescents 15 years old and younger [39].

Differences in reported prevalence among studies may be attributed to the use of different definitions and classifications which can complicate comparison and integration of research findings [40]. In Page et al.’s study among learners in formal high schools, respondents were asked a single-item question on whether they have ever attempted suicide whereas the current study attempted to distinguish between different types of attempts using several questions [35]. For these reasons, researchers and organizations developed recommended terminologies, which resulted in the US Centers for Disease Control and Prevention creating a guideline which recommended uniform definitions for suicidal behavior [41]. These definitions, which distinguish different types of suicidal ideation and behaviors, were adapted from the Columbia Suicide Severity Rating Scale, which was used to identify suicidal ideation and behaviors among the present study’s respondents.

The high prevalence of lifetime suicidal ideation and behavior reported in the present study may also be due to learners’ experience of triggers such as adverse life experiences. Early adverse life experiences, such as abuse and collapse of family ties, are identified risk factors for adolescent suicidal ideation and attempts [42–44]. Majority of the learners experienced such kinds of life experiences, with broken families and poor relationships with parents being mentioned frequently during interviews with both the teachers and learners. Furthermore, in this study, some learners reported bullying as the reason why they stopped attending formal school. Bullying and violence among peers are also risk factors for suicidal behavior [45, 46], with a causal association between bullying victimization and depression, suicidal ideation, and behaviors already established [47]. Majority of the learners also come from families living in impoverished communities, which increases the risk of suicidal behavior [48].

Progression of suicidal ideation to suicidal behavior is also influenced by the presence of moderators such as having access to means of suicide, increased capability to attempt suicide (e.g., high pain tolerance level), exposure to suicide, and impulsivity. In this study, the total number of lifetime and past month one-time attempts was almost the same as two or more attempts, except for non-fatal attempts wherein twice or more attempts were higher. History of previous attempts has been identified as strong predictors of completed suicide and subsequent suicide attempt among adolescents [49, 50].

The present study also found that the learners generally had less permissive attitudes towards suicide. One possible explanation for this finding is the religious background of the respondents. Majority of Filipinos and the respondents of this study are Roman Catholic Christians. Catholicism strictly forbids and does not condone suicide among its followers [51]. This tenet is also supported by the interview data, with the learners believing that suicide is a sin against God. Fear of going to hell as a consequence of committing suicide was also consistently mentioned throughout the interviews, with learners specifying this reason why they have not engaged in suicidal behavior.

The second factor, “Suicide is a process,” has also been observed in a previous study among college students in Korea [52]. In contrast to Korean college students, the present study’s learners who endorsed suicidal ideation and/or behaviors during their lifetime had a significantly lower score on this factor (less agreement). This might have an implication on intervention and prevention strategies, since impulsivity enables suicidal behavior [8]. The findings on learners’ attitudes towards suicide provide evidence for possible suicide prevention programs. Attitudes towards suicide can potentially be used to screen for potential suicidal ideation or behavior since learners may be more comfortable to answer these questions. However, further research focusing on the extent of how attitudes can be used to predict suicidal ideation and behavior must be done. Additionally, learners’ attitudes towards suicide can be used to create suicide prevention strategies focusing on attitude modification [53].

Understanding how these factors enable or prevent the development of suicidal ideation and progression to suicidal behavior is important for developing intervention strategies. Data from the qualitative interviews extended an understanding of the learners’ suicidal ideation and behavior and attitudes towards suicide. Furthermore, it also provided an understanding of practices and teacher-learner interactions within the ALS which can influence adolescent suicidal ideation and behaviors. These practices and interactions present in the ALS addressed moderators identified by the Integrated Motivational-Volitional model as potential targets for suicide intervention.

One of the objectives of the ALS is to impart life skills such as problem-solving and coping skills, and the teachers’ teaching philosophy clearly reflects this. In a study conducted in the Central Philippines region, the ALS was effective in developing the life skills of the learners, with problem-solving and decision-making attained to a great extent. Coping with emotions and stress were also achieved to some extent [54]. Problem-solving and coping skills are key threat-to-self moderators which, if present, can result to the development of feelings of entrapment.

Motivational moderators, which affect the likelihood of entrapment developing into suicidal ideation, were also addressed in the ALS. In the ALS classroom, belongingness, social support, and motivation were clearly emphasized through teacher-learner interactions. Previous studies which measured emotional health, suicidal behavior, and school connectedness demonstrated that higher school connectedness translated to reduced negative emotional health and suicidal behavior [55, 56]. Being part of a community or network with enhanced means of communications and sources of information was also highlighted. A more open communication between learners and teachers can result to improved capability to detect psychological distress or a higher likelihood that an adolescent will disclose suicidal behavior, and this is an important step since disclosure is the first step in the help-seeking process [57]. By being more connected, teachers were better able to address warning signs and secure learners’ safety. The close relationship within cliques as described by the learners also provides a good opportunity for fellow learners to provide support or to share concerns with a responsible adult. Additionally, the availability of other school personnel and community-based services are strengths in the ALS since it provides opportunities for better recognition, referral, and management of learners with suicidal ideation and behavior. However, the strategy of escalating learner concerns to experts can also possibly contribute to reduce help-seeking, as observed among students who self-harmed [13].

The strong religiosity and spirituality exhibited by the respondents can also have both positive and negative effects on mental health and suicidal ideation and behaviors. Strong religious beliefs and engaging in religious practices promote happiness and give meaning in life [58]. However, to the extent that religion acts as a prophylactic against suicidal ideation and behavior—it could be seen in some cases as decreasing the need for psychological or emotional counseling. In other studies conducted among adolescents, highly religious adolescents reported a lower probability of seeking help from mental health professionals [59], or some preferred to seek help from religious clergy [60].

On the other hand, moderators which could enable progression of suicidal ideation to behaviors were addressed by securing learners’ safety through preventive and response measures. This measure is important, since the study also found multiple attempts among the learners. Removing access to means for suicide, one of the measures mentioned in the interviews, has been identified as a key component of suicide prevention [61].

The ALS can also be an integral component in strengthening community-based mental health and social care services. This is in line with the World Health Organization’s Mental Health Action Plan for 2013 to 2020, which highlights a multi-sectoral and community-based approach for mental health [62]. Additionally, the newly ratified Mental Health Act of the Philippines legally mandates the Department of Education to integrate strategies promoting mental health in educational institutions [63].

Republic Act No. 11306, or the Mental Health Act of the Philippines, requires all educational institutions to integrate mental health in the curriculum of all educational levels. Several modules currently being used in the ALS already discuss concepts related to mental health, such as managing stress and preventing risky health behaviors. The availability of these modules provides an opportunity for educators to strengthen the integration of mental health in the curriculum. In Thailand, a project on improving psychosocial health of adolescents used curricular integration of mental health concepts to improve psychosocial well-being. This process was done in consultation with curriculum developers and mental health experts and by taking advantage of the existing curriculum and involving local stakeholders in the process, the model was successful in promoting capacity building and ownership in the community [64].

RA 11306 also endorses the promotion of mental health and well-being in schools. According to the UNICEF Framework for rights-based, child-friendly educational systems and schools, teachers play a significant role in creating effective and inclusive classrooms [65]. This study found that ALS teachers are the first to respond to concerns, including endorsement of suicidal ideation and behaviors, posed by the learners. However, specific or formal training on mental health was a need identified among the teachers and they have expressed their desire to strengthen their skills in interacting with learners with suicidal ideation and behaviors. The same findings were reported in a study conducted among US [66] and Canadian teachers [67], where lack of adequate staff training was identified as a possible barrier to provide necessary services to learners. Results of this study also indicate that both the teachers and learners still hold stigmatizing attitudes towards suicide. Stigma is a major barrier for help-seeking, since stigmatizing beliefs and attitudes of both the teacher and fellow learners can minimize opportunity for disclosure and influence learners’ help-seeking behavior. By improving mental health literacy of teachers, intention to help learners can be increased and stigma can be reduced [68]. Since teachers are in the front line for providing learners’ needs, it is necessary that they are equipped with the knowledge and skills needed to identify learners with suicidal ideation and behavior, how to interact with them, and link them with appropriate mental health services. Furthermore, teachers can also benefit from a training focused on basic mental health concepts and how to effectively communicate with learners with suicidal ideation and behaviors. Pre-service or in-service trainings can also be maximized to deliver mental health literacy trainings for teachers. There is supporting evidence that these trainings can impact how teachers can address learner needs [69]. Additionally, teacher training must also emphasize how to promote well-being and positive psychology within the classroom.

The present study also highlights the need to forge partnerships with mental health experts, another directive stipulated by RA 11306. Some teachers recognized that they can only provide support to learners to some extent, and that addressing suicidal ideation and behaviors warrants the assistance of guidance counselors or mental health experts. Rothi, Leavey, and Best also reported the same findings among schoolteachers in the UK [70]. ALS teachers identified school guidance teachers or guidance counselors as the school staff where they can refer learners with suicidal ideation and behaviors. However, some expressed concerns about the guidance teacher or guidance counselor’s capability to address these concerns, since they also must cater to the needs of the students in formal school. The link between the ALS and other community-based services can also be further maximized by connecting these services to the existing referral system. The schools can benefit from establishing networks with mental health experts from government institutions, non-government organizations, or faith-based organizations who can provide professional services to learners.

The findings of the present study must be interpreted with several points to consider. Since the results were generated using data from a cross-sectional study, the study cannot identify causation between the variables measured. Despite attempts to improve recruitment, the quantitative portion of the study had a small sample size. Some of the learners who were present during the study orientation were always absent during the researchers’ visits to collect data in the study sites. Others failed to secure parental consent despite constant reminders during the researchers’ visits to the study sites. More representative results can be obtained if the response rate is improved. Other recruitment methods such as the use of social media or other technology-based strategies can be employed to improve response rate. For example, future researchers can start advertising the study in social media sites a few months prior to data collection so that respondents will be more familiar with the study and its objectives [71]. Finally, adolescents in other out-of-school settings, such as those in conflict with the law, may have experiences other than those described in this study and are possible avenues for further research.

## Conclusion

This study showed that suicidal ideation and behaviors are prevalent among adolescent ALS learners. This study also demonstrated a significant difference in attitudes towards suicide and sociodemographic characteristics between learners who experienced suicidal ideation or behaviors and those who did not. It also suggests that the school psychosocial environment, through existing social norms and learner-teacher interactions, can potentially prevent progression of suicidal ideation to behavior, influence help-seeking, and promote mental health among learners. The results also confirm the need for suicide intervention strategies and teacher training in the ALS, with specific focus on proper communication with learners endorsing suicidal ideation and behaviors, identification of warning signs, and ways to intervene when the need arises.

## Abbreviations

A&E: Accreditation and Equivalency
ALS: Alternative Learning System
ATTS: Attitudes Towards Suicide
C-SSRS: Columbia Suicide Severity Rating Scale
DSWD: Department of Social Welfare and Development
GSHS: Global School-based Health Survey
IMV: Integrated Motivational Volitional Theory
UNESCO ISCED: United Nations Educational, Scientific and Cultural Organization International Standard Classification of Education
